# Organic superstructure microwires with hierarchical spatial organisation

**DOI:** 10.1038/s41467-021-22513-5

**Published:** 2021-04-15

**Authors:** Ming-Peng Zhuo, Guang-Peng He, Xue-Dong Wang, Liang-Sheng Liao

**Affiliations:** 1 Institute of Functional Nano & Soft Materials (FUNSOM), Jiangsu Key Laboratory for Carbon-Based Functional Materials & Devices, Soochow University, Suzhou, Jiangsu P. R. China; 2 Institute of Organic Optoelectronics, JITRI, Wujiang, Suzhou, Jiangsu P. R. China

**Keywords:** Self-assembly, Nanowires

## Abstract

Rationally designing and precisely constructing the dimensions, configurations and compositions of organic nanomaterials are key issues in material chemistry. Nevertheless, the precise synthesis of organic heterostructure nanomaterials remains challenging owing to the difficulty of manipulating the homogeneous/heterogeneous-nucleation process and the complex epitaxial relationships of combinations of dissimilar materials. Herein, we propose a hierarchical epitaxial-growth approach with the combination of longitudinal and horizontal epitaxial-growth modes for the design and synthesis of a variety of organic superstructure microwires with accurate spatial organisation by regulating the heterogeneous-nucleation crystallisation process. The lattice-matched longitudinal and horizontal epitaxial-growth modes are separately employed to construct the primary organic core/shell and segmented heterostructure microwires. Significantly, these primary organic core/shell and segmented microwires are further applied to construct the core/shell-segmented and segmented-core/shell type’s organic superstructure microwires through the implementation of multiple spatial epitaxial-growth modes. This strategy can be generalised to all organic microwires with tailored multiple substructures, which affords an avenue to manipulate their physical/chemical features for various applications.

## Abstract (web summary)

Rationally designing and precisely constructing the dimensions, configurations and compositions of organic micro- and nanomaterials are key issues in material chemistry, but remain challenging. Here, the authors realize the fine synthesis of organic superstructure microwires via a hierarchical epitaxial-growth approach.

## Introduction

The precise synthesis of one-dimensional (1D) nanowires with accurate spatial organisation have received wide attention for its fundamental scientific understandings and industrial applications^1–4^. To date, the fine synthesis of inorganic or metal nanowires has significantly achieved the precise control of structure, size, and components using various methodologies and mechanisms^5,6^. Representative cases include zinc oxide nanowire arrays with tuneable size distribution prepared via physical vapour deposition^7^ and lead sulphide nanowires resembling pine tree based on a dislocation-driven nanowire growth mechanism^8^. Notably, complex micro/nanostructures generally demonstrate superior physical/chemistry properties, such as enhanced charge carrier density on the core/shell interfaces^9,10^ and spatially dependent multicolour emission in segmented structures^11^, which render them promising candidates for high-performance optoelectronic applications^12^. In contrast, the fine synthesis of complex micro/nanostructures with dissimilar components/substructures presents greater challenges^13,14^, which must be overcome to meet practical nanotechnology requirments^15–17^. For example, Cahoon and co-workers^18^ demonstrated geometric superlattices Si nanowires with designed nanophotonic properties using a complex vapour-liquid-solid growth process.

To meet the ever-increasing practical demand for flexible and lightweight nanotechnological functions, organic nanomaterials present alternative opportunities^19^. Recently, organic semiconductor micro/nanostructures have become a hot topic because of the variety and flexibility of their molecular design and synthesis, tuned physical/chemical properties, and low-cost large-area fabrication^20,21^. However, notably, organic micro/nanostructures, particularly organic superstructure nanowires composed of dissimilar materials and hierarchical structures, have been rarely developed by currently-investigated material systems. The difficult manipulation of the homogeneous/heterogeneous-nucleation process and the complex epitaxial relationships of different material combinations^5,22,23^ are barriers that need to be overcome for the precise construction of organic superstructure nanowires. The precise control of the morphological dimensions of organic micro/nanostructures has been achieved, which depends on the rational modulation of homogeneous/heterogeneous-nucleation via adjustment of the experimental conditions, such as the concentration of the secondary component^24,25^, the solution temperature^26,27^, the superhydrophobic feature^28^, and the regional roughness of the seed surface^29^. Directed by these successes, the manipulation of spatial homogeneous/heterogeneous-nucleation is a feasible and versatile but underdeveloped strategy for the construction of organic superstructure nanowires in the elaborate regulation of the fine structure/component.

**Fig. 1:**
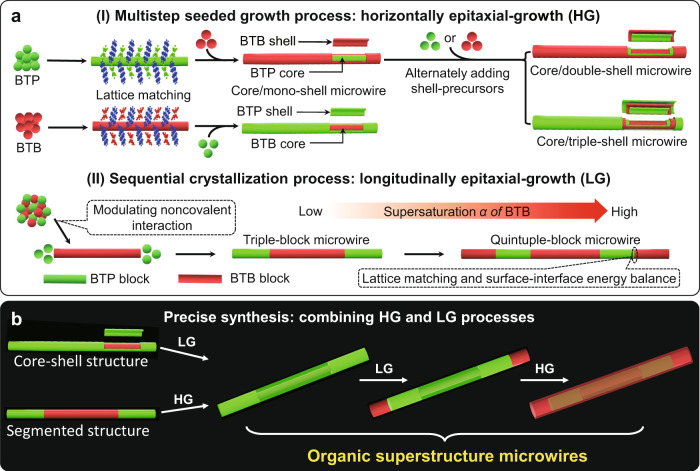
Illustration of the epitaxial-growth for organic superstructure microwires. **a** Schematic illustrations: (I) the horizontal epitaxial-growth mode for the organic core/shell microwires via a multistep seeded growth process and (II) the longitudinal epitaxial-growth mode for the organic multiblock microwires via a sequential crystallisation process. The red-emissive and green-emissive cartoon spheres represent the BTB and BTP cocrystal, respectively. Their chemical structures were shown in Supplementary Fig.1. **b** Schematic illustration of the combination of the horizontal and longitudinal epitaxial-growth modes for the organic superstructure microwires.

Herein, we propose a fine synthesis approach for organic superstructure microwires with hierarchical spatial organisation that combines lattice-matched epitaxial growth along both horizontal and longitudinal directions (Fig. 1b). For two types of primary organic heterostructure microwires, the core/shell microwires can be prepared via the horizontal epitaxial-growth process along the radial direction of pre-existing crystal, and the segmented microwires can be fabricated via the longitudinal epitaxial-growth process along the axial direction of pre-existing crystal (Fig. 1a). Typically, as-prepared benzo[ghi]perylene-1,2,4,5-tetracyanobenzene (BTB) cocrystal microwires are added as seeded cores into a benzo[ghi]perylene-tetrafluoroterephthalonitrile (BTP) cocrystal-saturated supernatant as a shell-layer solution, resulting in the horizontal epitaxial-growth of BTP on the core surface that formed the BTB/BTP core/shell microwires. Furthermore, the interchanging of components and multi-shell structures in core/shell microwires can be realised by rationally choosing the core/shell-component and alternately adding shell-precursors, respectively. The ordered self-assembly and longitudinal epitaxial-growth is a result of BTP and BTP lattice energies (|*E*_BTB_ = −66.57 kcal mol^−1^ | > | *E*_BTP_ = −60.15 kcal mol^−1^ | ; see Table S1), which contribute to the formation of segmented microwires: triple- and quintuple-block microwires. Significantly, by elaborately combining longitudinal/horizontal epitaxial-growth processes, we are able to synthesise various organic superstructure microwires with multi-diverse core/shell substructures and segmented heterostructures (Fig. 1b). For example, following hierarchical epitaxial-growth, the core/shell organic microwires can be further applied to construct core/shell-segmented-type organic superstructure microwires, whereas the segmented-core/shell type organic superstructure microwires can be fabricated based on the triple-block microwires as initial building blocks. This proposed spatial epitaxial growth is a flexible and versatile approach to the design and construction of organic superstructures with tailored multi-level heterostructures.

## Results

### Horizontal epitaxial-growth mode for the core/shell microwires with component interchange and multi-shell structure

**Fig. 2:**
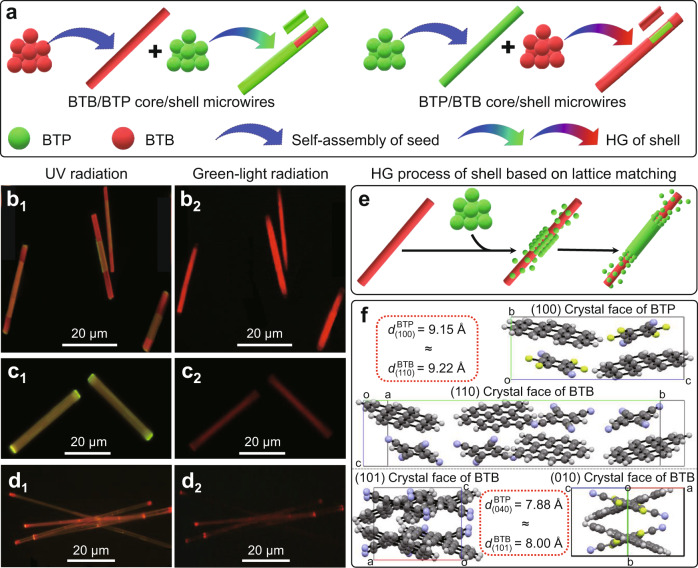
Core/shell microwires with component interchange. **a** Schematic representation of the horizontal epitaxial-growth approach for the organic core/shell microwires with component interchange. FM images of **b** BTB microwires with partly covering by BTP, **c** BTP/BTB microwires, and **d** BTP/BTB core/shell microwires with the scale bars of 20 μm. The microwires in (**b**_1_, **c**_1_, and **d**_1_) were excited with UV light (*λ* = 330–380 nm) from a mercury lamp. Although the microwires in (**b**_2_, **c**_2_, and **d**_2_) were excited with green light (*λ* = 500–550 nm) from a mercury lamp. **e** Schematic representation of horizontal epitaxial-growth process for the formation of BTB/BTP core/shell microwires. **f** Molecular arrangement and orientation at the junction of core/shell structure.

The green-emissive BTP and red-emissive BTB cocrystal microwires were prepared by a facile solution-evaporation method^30–33^, as illustrated in Supplementary Figs 1–3. Through a multistep seeded growth method, the BTP and BTB can be integrated into the core/shell microwires with component interchange after seeded self-assembly and a shelly horizontal epitaxial-growth process (Fig. 2a). After adding BTP supersaturated solution as shell solution into the growth solution system of the BTB microwire seeded core, the mixed solution is quickly dropped on a quartz plate. The as-prepared microwires display a green-emissive layer partly depositing on the centre of red-emissive core (Fig. 2b_1_). Only the core part was excited by green light, suggesting that the horizontal epitaxial-growth process of the shell layer is activated at the seed-core centre part via the heterogeneous-nucleation process (Fig. 2e). The red microwires with incomplete cladding by green-emissive BTP or little blue-emissive Benzo[ghi]perylene (Supplementary Fig. 4a, b) further confirm the core/shell structure. The BTB/BTP core/shell microwires with smooth surface were successfully prepared after the horizontal epitaxial-growth process of the BTP on the surface of pre-existing BTB core microwires, which display yellow emission with UV-light excitation and red emission with green light excitation (Fig. 2c, Supplementary Fig. 4c, d). As shown in Fig. 2d, these as-prepared organic heterostructure microwires display a red-emissive layer covering on the green-emissive microwires with the UV-light excitation, and red-emissive microtubes with the nonemissive inner part excited by the green light. The spatially resolved PL spectra of these two kinds of heterostructure microwires include the red emission (*λ*_2_ = 600 nm) from BTB cocrystal and the green emission (*λ*_1_ = 515 nm) from BTP cocrystal (Supplementary Fig. 5), which suggests the formation of core/shell structure with the interchanging component^21^. Moreover, from the crystal-structural aspect, the X-ray diffraction (XRD) patterns of the two core/shell microwires are composed of the characteristic diffraction peaks of both BTP and BTB (Supplementary Fig. 6). Combining the scanning electron microscopy (SEM) images (Supplementary Fig. 7), further verifies the formation of the crystalline core/shell microwires with smooth surface. In addition, other organic solvent systems and the high temperature of the stock solution will impede the formation of the core/shell structure, as indicated by the FM image in Supplementary Fig. 8.

The horizontal epitaxial-growth mechanism of core/shell structure was elucidated by the molecular parking arrangements at the heterojunction^24^. As shown in Fig. 2f, the two-component molecules in BTP and BTB cocrystals adopt face-to-face parking modes with analogous molecular arrangements at the heterojunction, which is beneficial for epitaxial-growth between BTP and BTB for preparing organic heterostructure. Furthermore, the crystal-face of the two charge-transfer (CT) cocrystals at the junction have a low lattice mismatch ratio (*f*) of 0.8% ($d^{(100)}_{\rm{BTP}}$  = 9.15 Å ≈ $d^{(110)}_{\rm{BTP}}$ = 9.22 Å, Supplementary Table 2) and 1.5% ($d^{(101)}_{\rm{BTP}}$ = 8.00 Å ≈ $d^{(040)}_{\rm{BTP}}$ = 7.88 Å, Supplementary Table 2), which facilitates the epitaxial-growth between the BTP and BTB for preparing organic heterostructure. In contrast, the vertical epitaxial-growth process between the BTB and BTP from the branched structure is unable to realise, which is owing to the huge lattice mismatching at the vertical heterojunction (Supplementary Figs. 9–11). Therefore, the small lattice mismatching and the analogous molecular arrangements contribute to the preferential aggregation of the BTP on the surface of the BTB microwires, inducing the selective heterogeneous-nucleation and horizontal epitaxial-growth process for the formation of the core/shell structure.

**Fig. 3:**
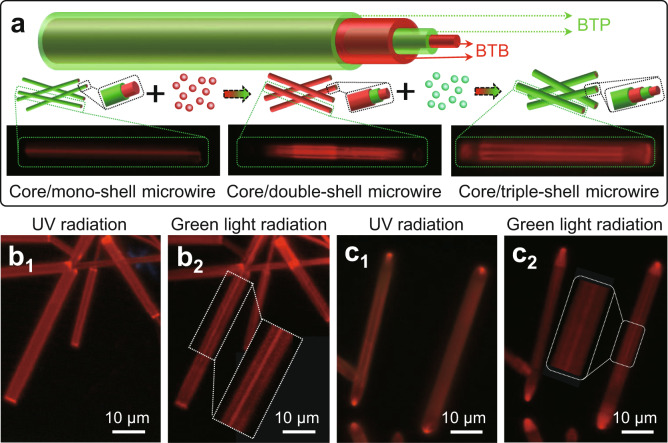
Core/multi-shell organic microwires. **a** The horizontal epitaxial-growth approach for the core/multi-shell organic microwires. **b** FM images of the core/double-shell organic microwires excited with **b**_1_ the UV light and **b**_2_ green light. **c** FM images of the core/triple-shell organic microwires excited with **c**_1_ the UV light and **c**_2_ green light. Scale bars of **b** and **c** are 10 μm.

According to the horizontal epitaxial-growth process, the well-defined core/double-shell and core/triple-shell microwires made up of BTP and BTB were rationally designed and constructed by alternately adding shell-precursors (Fig. 3a). The pre-existing BTB/BTP core/shell microwires were employed as seed for the horizontal epitaxial-growth of BTB, resulting in the BTB/BTP/BTB core/double-shell microwires. Remarkably, the Fluorescence microscopy (FM) images of core/shell microwires show an alternating strong/weak red-emissive phenomenon along the horizontal direction excited by green light, suggesting their multi-shell feature. As shown in Fig. 3b_1_, these as-prepared core/double-shell microwires present red emission with UV-light excitation. After changing the excitation from UV light to green light, the intermediate sandwich becomes nearly nonemissive, the strong red-emissive microwires and nanotubes with the same axes were found (Fig. 3b_2_), which indicates the formation of the core/double-shell structure. Following further horizontal epitaxial-growth of BTP shell, the BTB/BTP/BTB core/double-shell microwires transform into the BTB/BTP/BTB/BTP core/triple-shell microwires. These as-prepared core/triple-shell microwires display red emission excited by UV light (Fig. 3c_1_). Under green-light excitation, the phenomenon of the alternate distribution of nonemission and red emission appears along the horizontal direction (Fig. 3c_2_), which confirms the formation of a core/triple-shell structure. Therefore, the core/multi-shell organic microwires comprising BTP and BTB were finely prepared via the multistep horizontal epitaxial-growth processes by alternately adding the shell-precursors.

### Longitudinal epitaxial-growth mode for segmented organic microwires with tunable multi-blocks structure

**Fig. 4:**
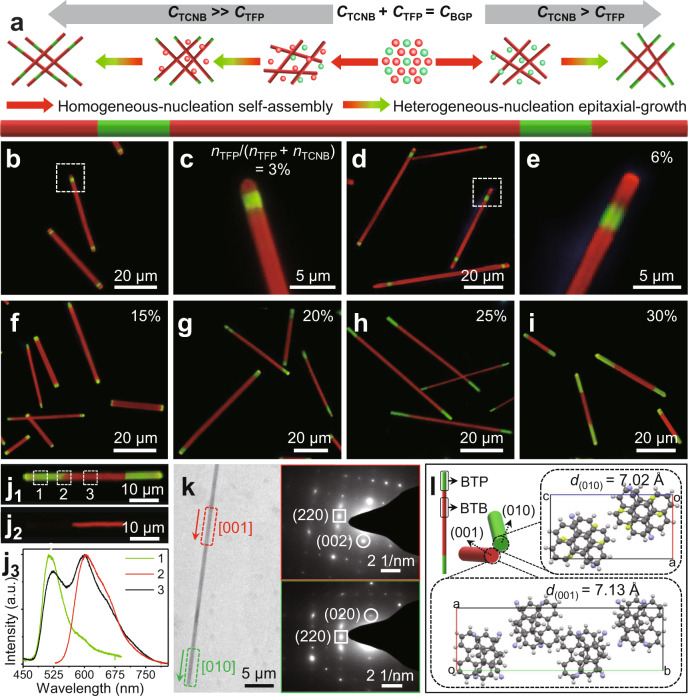
Segmented organic microwires with tunable multiblock structure. **a** The longitudinal epitaxial-growth mode for the segmented organic heterostructure microwires. **b**–**e** FM images of organic quintuple-block microwires prepared with the acceptor molecular ratio *η*_TFP_ of **b**, **c** 3% and **d**, **e** 6%. **f**–**i** FM images of organic triple-block microwires prepared with the acceptor molecular ratio *η*_TFP_ of **f** 15%, **g** 20%, **h** 25%, and **i** 30%. The scale bars of **b**, **d**, **f**–**i** and **c**, **e** are 20 and 5 μm, respectively. **j** FM images of one typical organic triple-block microwire with (**j**_1_) UV-light and (**j**_2_) green-light excitation; and (**j**_3_) the spatially resolved PL spectra corresponding to different locations marked domains in (**j**_1_). **k** TEM image of one typical organic triple-block microwire with a scale bar of 5 μm. SAED patterns at the right-upper and the right-below insets collected from the centre part (marked in red gridlines) and the end part (marked in green gridlines) in triple-block microwire, respectively. **l** Molecular arrangement and orientation at the junction of multiblock structure.

As illustrated in Fig. 4a, the triple-/quintuple-block organic heterostructure microwires including green-emissive BTP and red-emissive BTB have been successfully prepared through a longitudinal epitaxial-growth mode in the sequential crystallisation process. Based on the different levels of intermolecular interactions (| $E^{\rm{interaction}}_{\rm{BTP}}$ = −3.14 kcal mol^−1^ | > | $E^{\rm{interaction}}_{\rm{BTP}}$ = −2.84 kcal mol^−1^ | ) and lattice energies (|$E^{\rm{lattice}}_{\rm{BTP}}$ = −66.57 kcal mol^−1^ | > |$E^{\rm{lattice}}_{\rm{BTP}}$ = −60.15 kcal mol^−1^ | ) (Supplementary Table 1), the sequential homogeneous-/heterogeneous-nucleation, and the longitudinal epitaxial-growth were controllably modulated through the adjustment of BTP supersaturation by tuning molar ratio *η*_TFP_ of the electronic acceptor of tetrafluoroterephthalonitrile (TFP), combined with another electronic acceptor of 1,2,4,5-tetracyanobenzene (TCNB) ($\eta =\eta {\rm{TFP}}/(\eta {\rm{TFP}}+\eta {\rm{TCNB}})$). The functional block number and the corresponding block length ratio in organic segmented microwires can be elaborately adjusted by adjusting the *η*_TFP_. FM images of these as-prepared mutliblock organic microwires (Fig. 4b–i) display the well-defined segmented structures with alternating green-red emission under UV-light excitation. XRD patterns of triple-/quintuple-block mutliblock organic microwires (Supplementary Fig. 12) contain the characteristic diffraction peaks of both BTP and BTB microwires, which confirms their components of the high crystalline BTP and BTB^17^. With high TFP molar ratios *η*_TFP_ (12%~30%), the triple-block organic microwires with green-red-green segmented structure were finely fabricated via a sequential homogeneous-/heterogeneous-nucleation and a longitudinal epitaxial-growth. As indicated in Fig. 4f–i and Supplementary Fig. 13, after increasing the *η*_TFP_ from 12% to 30%, the length ratio between the green-emissive and red-emissive blocks demonstrates considerable enhancement. It shows that the length ratio of the green-emissive at the tips in the triple-block microwires is determined by the BTP supersaturation. Therefore, this longitudinal epitaxial-growth process supplies a strategy to controllably tune functional block length in the segmented structures.

In contrast, the further heterogeneous-nucleation and epitaxial-growth processes are triggered by decreasing the TFP molar ratios *η*_TFP_ to <12%, yielding the quintuple-block organic microwires with emission structure under UV-light excitation (Fig. 4b, d). The resolution FM images marked with white gridlines in Fig. 4b, d display a constant length ratio of the green-emissive block. An obvious enhancement in the length of the red-emissive block at the tips was found after increasing the TFP acceptor molar ratios *η*_TFP_ from 3% to 6%. It reveals that the segmented structure in the quintuple-block organic microwires also can be finely adjusting by the TFP molar ratios *η*_TFP_. After changing the excitation from UV light to green-light, the centre of the triple-block microwires still emit red light, whereas green emission of the end parts transfer into the nonemission state (Fig. 4j). As shown in Fig. 4j_3_, the spatially PL spectra of the end (marked as 1, *λ*_1_ = 515 nm) and the centre (marked as 2, *λ*_2_ = 600 nm) parts are in good consistence with those of BTP and BTB, respectively (Supplementary Fig. 2a). In addition, the PL spectrum of the heterojunction involves red emission (600 nm) from BTB and green emission (515 nm) from BTP. The optical results from the Fig. 4j confirm that the centre and end blocks in the triple-block organic microwires correspond to red-emissive BTB and green-emissive BTP, respectively.

In the sequential self-assembly process, the BTB molecules firstly were self-assembled into the seeded microwires driven by the high lattice energy and the strong CT interaction, then BTP molecules attached on the tips of BTB microwires, and epitaxially grown into triple-block microwires. The modulation of the fine structure of the heterostructure generally depends upon the concentration molar ratio between two acceptors^31^. With the *η*_TFP_ of 15%~ 3%, the BTP molecules were mostly self-assembled into the green-emissive block in the early stage. With the evaporation of the good solvent of dichloromethane, the concentration of BTB increased to the supersaturation sate again, which is due to the initial high ratio of BTB in the mixed solution. Then, the heterogeneous-nucleation and longitudinal epitaxial-growth processes of BTB are triggered to form another two extended red-emissive block at the tip of the triple-block microwires. In contrast, the quality of the remnant BTP is not enough to provide further heterogeneous-nucleation and longitudinal epitaxial-growth processes, leading to the formation of the quintuple-block microwires. With the *η*_TFP_ of 15%~30%, there are enough BTP molecules to have further heterogeneous-nucleation and longitudinal epitaxial-growth processes, which lead to the increasing length of the green-emissive blocks. Due to the considerable difference in the supersaturation between the BTP and BTB in the residual solution, the BTB is hardly to achieve the supersaturation state for the epitaxial-growth process on the tips of triblock microwires. Therefore, the high *η*_TFP_ is a key factor for the construction of the triblock microwires with long green-emissive blocks.

The SEM and transmission electron microscope images (Fig. 4k, Supplementary Figs. 14, 15) indicate that the smooth surface feature with high crystalline of these as-prepared mutliblock organic microwires, which are analogous to the BTP microwires, BTP microwires, and the core/shell microwires. As illuminated in Fig. 4k, the selected area electron diffraction (SAED) pattern of the marked central part (red gridlines) triple-block microwire exhibit an identical pattern as that of the crystalline BTB microwires cocrystal growing along the same direction of [001] (Supplementary Fig. 2c). Meanwhile, it was found that the SAED pattern of the marked end part (green gridlines) in triple-block microwire exhibit the identical pattern as that of the crystalline BTP microwires cocrystal growing along the same direction of [010] (Supplementary Fig. 2f). These structural results reveal that the heterojunction is between the (001) face of BTB and (010) face of BTP. Notably, these two different crystal faces demonstrate the same packing arrangement with face-to-face mode (Fig. 4l) and have a low lattice mismatch ratio (*f*) of 1.5% ($d^{(001)}_{\rm{BTB}}$  = 7.13 Å ≈ $d^{(010)}_{\rm{BTB}}$ = 7.02 Å, Supplementary Table 2). The packing arrangements along the [001] direction in BTB crystal and the [010] direction in BTP crystal are also the same as verified in Supplementary Fig. 16. Hence, the favourable lattice matching and the analogous packing arrangement are beneficial for a facile longitudinal epitaxial-growth of BTP at the junction along the *b* axis on the tips of BTB microwires.

### The hierarchical epitaxial-growth approach for organic superstructure microwires

**Fig. 5:**
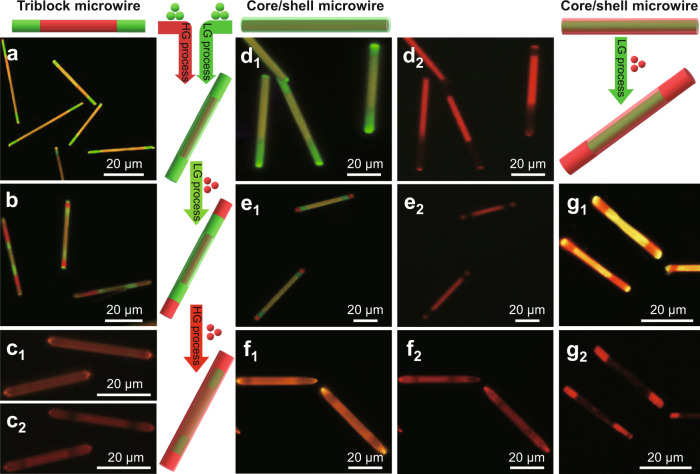
Rational design and fine synthesis of organic superstructure microwires. FM images of segmented-core/shell **a** type-I, **b** type-II, and **c** type-III organic superstructure microwires. FM images of core/shell-segmented **d** type-I, **e** type-II, **f** type-III, and **g** type-VI organic superstructure microwires. The scale bars are 20 μm. The organic superstructure microwires as shown in **a**, **b**, **c**_1_–**g**_1_, and **c**_2_–**g**_2_ were excited with the UV light and green light, respectively.

Combining horizontal and longitudinal epitaxial-growth processes for rational design and accurate synthesis of the organic superstructure microwires made up of both segmented and core/shell structures have been regarded as a proof of concept and successfully achieved. In order to realise the segmented-core/shell type organic superstructure microwires, these as-prepared triple-block microwires were added as seeds into a saturated supernatant of BTP solution for horizontal epitaxial-growth process, leading into the final organic superstructure (segmented-core/shell type-I) microwires. The centre region in these segmented-core/shell type-I organic microwires displays yellow emission with UV-light excitation (Fig. 5a and Supplementary Fig. 17a) and red emission with green-light excitation (Supplementary Fig. 17c), which are in accordance with those of the BTB/BTP core/shell microwires (Fig. 2c), suggesting the formation of the core/shell structure. Moreover, the green-emissive end regions in these segmented-core/shell type-I microwires turn into nonemissive regions after changing the excitation from UV light to green light, which is consistent with that on the end tip of the BTP-BTB-BTP triple-block microwires, implying the formation of segmented structure. As shown in Supplementary Fig. 18, the spatially PL spectra of the end (marked as 1, *λ* = 515 nm) and the centre (marked as 2, *λ* = 600 nm) parts are in good consistency with those of BTP and BTB, respectively. And the spatially PL spectrum of the heterojunction (marked as 3) also exhibits the green emission (515 nm) from the BTP and red emission (600 nm) from BTB. The above results clarify the realisation of the integrated segmented-core/shell type superstructure. Furthermore, the longitudinal epitaxial-growth process was introduced after quickly adding a saturated supernatant of BTB solution into the above balanced horizontal epitaxial-growth system. Then, the segmented-core/shell type-II organic microwires with two red-emissive BTB block epitaxially growing at the tips of segmented-core/shell type-I microwires were obtained (Fig. 5b). Interestingly, the segmented-core/shell type-II organic microwires can also be added as seed into a saturated supernatant of BTB solution, forming the segmented-core/shell type-III organic microwires. As illustrated in Fig. 5c, the segmented-core/shell type-III microwires display the uniform yellow emission with UV-light excitation and red–dark alternant segmented structure with green-light excitation. It confirms the horizontal epitaxial-growth process of the BTB on the surface of the segmented-core/shell type-II microwires for forming the core/double-shell structure.

Likewise, the core/shell microwires also can act as the seed for fine synthesis of another organic superstructure (core/shell-segmented type) microwires via further longitudinal or horizontal epitaxial-growth process. After adding a saturated supernatant of BTP solution into the balanced epitaxial-growth system of BTB/BTP core/shell microwires, the core/shell-segmented type-I microwires were controllably prepared, which present a green-yellow-green triple-block structure with the UV-light excitation (Fig. 5d_1_). Although the centre and the end parts, respectively, become strong red emission and nonemission regions excited by green light (Fig. 5d_2_). After increasing the shell thickness, the green–yellow–green triple-block structure showing in the as-prepared core/shell-segmented type-I microwires changes to the whole green-emissive structure with UV-light excitation (Supplementary Fig. 17a, b). Meanwhile, the corresponding emission intensity of nonemissive-red-nonemissive triple-block structure become weak with green-light excitation (Supplementary Fig. 17c, d), which is attributed to the shielding effect of the shell layer for the core part. These results verify that the longitudinal epitaxial-growth of the BTP at the ends of core/shell microwires for the fabrication of core/shell-segmented type superstructure. The core/shell-segmented type-II microwires were successfully achieved via a longitudinal epitaxial-growth process of BTB on the tips of the core/shell-segmented type-I microwires, which present red–green–yellow–green–red and red–dark–red–dark–red quintuple-block structure with UV-light and green-light excitation (Fig. 5e), respectively. Combining the yellow emission of the centre region turning into red emission after changing the excitation from UV light to green light, these results verify the formation of the core/shell structure at centre part and segmented structure at the end part. Finally, the core/shell-segmented type-III microwires were controlled prepared through a horizontal epitaxial-growth of BTB on the surface of the core/shell-segmented type-II microwires. The yellow emission of these as-prepared core/shell-segmented type-II microwires turns into red–dark–red–dark–red segmented structure after changing the excitation from UV light to green light (Fig. 5f), which is analogous that in the segmented-core/shell type-III microwires (Fig. 5c). Remarkably, the core/shell-segmented type-VI organic superstructure microwires were controllably fabricated via a longitudinal epitaxial-growth process by adding the saturated supernatant of BTB solution to the balanced epitaxial-growth system of BTP/BTB core/shell microwires. The core/shell-segmented type-VI microwires display the red–yellow–red triple-block structure under the UV-light excitation (Fig. 5g_1_). In contrast, the corresponding centre part becomes a red-emissive tube with a nonemissive core, and the end parts remain the red emission excited by green light (Fig. 5g_2_). It illustrates that BTB block epitaxially grows on the tip of BTP/BTB core/shell microwires, leading to a core/shell-segmented type superstructure. Therefore, the multiple horizontal/longitudinal epitaxial-growth processes were successfully employed to integrate multiple core/shell and segmented heterostructures into these controlled organic superstructure microwires.

### Organic superstructure microwires for multiple input/output optical logic gate

**Fig. 6:**
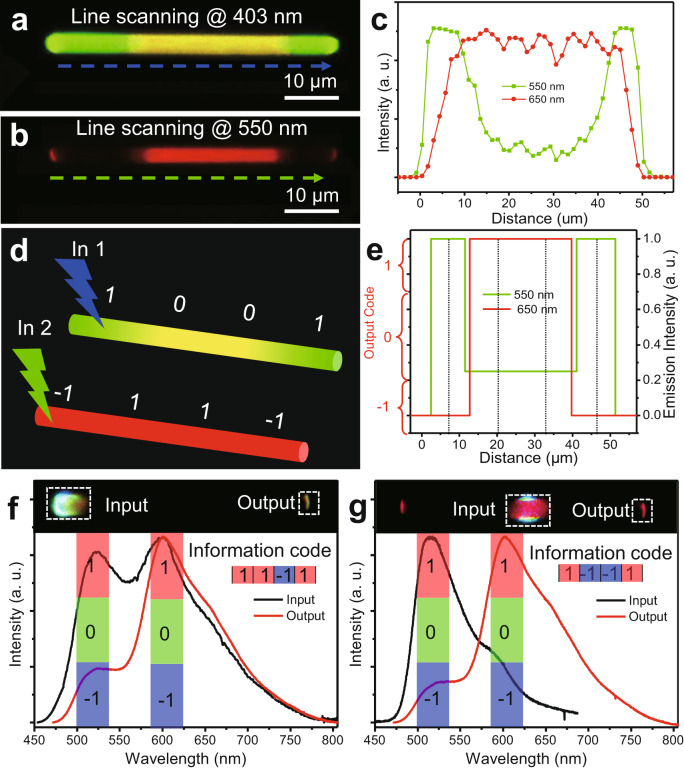
Organic superstructure microwires for optical logic gate. **a**, **b** FM images of individual segmented-core/shell type-I organic microwires excited by the **a** UV light and **b** green light. The scale bars are 10 μm. **c** The corresponding plots of the spatially emissive intensity as a function of the distance *d* along the wire axis. **d**, **e** Schematic diagram of **d** the luminescent barcode and **e** simulated decoding oscillography depended on the information on **a**–**c**. **f**, **g** Spatially resolved PL spectra collected from input and output channels after exciting with a laser beam (*λ* = 375 nm) at the end part **f** and the centre part **g**. Inset: the corresponding FM image.

The spatially segregated multicolour emission of the organic superstructure microwires prompted us to perform a variety of potential photonic applications, as in the case of a photonic transistor, optical logic gate, and wavelength converter realised by organic triblock microwire^34–36^. Therefore, a prototypical optical logic gate device based on an individual segmented-core/shell type-I organic superstructure microwire was proposed. Under UV irradiation, the segmented-core/shell type-I microwire shows green-yellow-green striped multicolour emissions along the wire axis (Fig. 6a). When the excitation changes from UV light to green light, only the core at the centre location emits an intensive red light (Fig. 6b). The PL spectra of the distal and the middle sections are mostly consistent with those of the BTP microwires and BTB/BTP core/shell microwires (Supplementary Fig. 18), demonstrating an integrated segmented-core/shell superstructure. As shown in Fig. 6c, the emissive intensities at different locations along the single segmented-core/shell type-I microwire and the dissimilar excited wavelength were treated like the optical encoding and decoding signals, respectively. The self-assembled organic segmented micro/nanostructure of the laser materials would demonstrate a great advantage in overcoming the obvious overlap between different emissions at the interface as shown in Fig. 6c. Furthermore, the relative intensity has been defined into three zones, which are corresponding to the optical signal of “1”, “0”, and “−1”, as shown in the stimulated signal oscillography (Fig. 6e). As a result displayed in Fig. 6d, the individual segmented-core/shell type-I organic microwire displays a series of information code of 1 0 0 1 and −1 1 1 −1 reading from the same four positions with the UV-light and green-light excitations, respectively.

Furthermore, the spatially dependent multicolour emission feature of the segmented-core/shell type-I microwire can be applied to construct the optical logic gate with multiple input/output channels. The optical-loss coefficients of BTP and BTB microwires (*R*_BTP_ = 0.021 dB μm^−1^ and *R*_BTB_ = 0.044 dB μm^−1^) were calculated out by accurately shifting the excitation laser spots along the wire axis (Supplementary Fig. 19a–f)^37^. In contrast, the BTB/BTP core/shell microwires show a two-channel optical waveguide information, as presented in Supplementary Fig. 19g-i. Moreover, the relative intensity of PL at 525 or 600 nm has been defined into three equational zones, corresponding to optical signal of “1”, “0”, and “−1”, respectively (Fig. 6f, g). When an alternative tip is excited in Fig. 6f, the photon propagation to the other tip demonstrates a conspicuous intensity decrease in the PL peak at 515 nm, resulting in the information code of 1 1 −1 1. Compared with the excited position at the middle sections (Fig. 6g), photon propagation to the other tip demonstrates an energy transfer from green to red emission (600 nm), giving an information code of 1 −1 −1 1. Owing to the synergetic effect of integrating the core/shell and segmented heterostructures, the as-prepared organic superstructure microwires demonstrate spatially segregated multicolour emission and anisotropic optical properties for photon transfer, realising an optical logic gate with multiple input/output channels.

## Discussion

In summary, a variety of organic superstructure microwires incorporating accurate components and substructures were successfully fabricated via a hierarchical epitaxial-growth approach with the simultaneous introduction and modulation of horizontal and longitudinal epitaxial-growth modes. First, the horizontal epitaxial-growth mode was applied to construct the core/shell microwires, with an elaborate control in the structural component or shell-layer number via an alternating shell-precursors addition process. Likewise, rational regulation of the component supersaturation was used to construct segmented microwires with precise modulation of either the block number or the length ratio via the longitudinal epitaxial-growth mode based on anisotropic lattice energy and intermolecular interaction. By adjusting the experimental conditions of the species or the order in which the materials were added, the organic superstructure microwires were precisely constructed by combining the multiple spatial epitaxial-growth modes. This was attributed to the high chemical/structural compatibility for heterogeneous-nucleation crystallisation and the epitaxial-growth processes. Furthermore, owing to anisotropic optical characteristics, these as-prepared organic superstructure microwires were employed for luminescent encoding/decoding and nanoscale multiple input/output optical logic gate. This proposed strategy provides new insight into the precise construction of organic superstructure microwires with hierarchical heterostructures and desired spatial configurations.

## Methods

### Primary self-assembly of organic BTP and BTB cocrystal microwires

The organic BTB and BTP microwires were obtained via the primary self-assembly process by a solution-evaporation method.1, 2 Typically, 0.1 mmol BGP and 0.1 mmol TFP were each dissolved into 10 mL DCM at room temperature to obtain the BTP stock solution with concentrations of 10.0 mmol L-1. Second, the BTP stock solution was added to 20 mL ethanol. The mixture was then dropped onto a quartz substrate. The formation of BTP microwires were observed was achieved after the solvents completely evaporated. Likewise, the 10 mL BTB stock solution, which composes of BGP and TCNB with the same concentrations of 10.0 mmol L^−1^ in DCM at room temperature, was added to 20 mL ethanol. Then, the mixture was directly dropped onto a quartz substrate, and the formation of BTP microwires was observed after the solvents completely evaporated.

### Horizontal epitaxial-growth mode for the organic core/shell microwires

The multistep seeded growth process was applied to prepare the organic core/shell microwires via a horizontal epitaxial-growth process. In a typical experiment, 0.2 mmol BGP, 0.2 mmol TCNB were first dissolved into 10 mL DCM to obtain the BTB stock solution with concentrations of 20 mmol/L at room temperature. As well as 0.2 mmol BGP, 0.2 mmol TFP were dissolved into 10 mL DCM to obtain the BTP stock solution with concentrations of 20 mmol/L at room temperature. Second, the 10 mL BTB stock solution was added into the 20 mL mixed solution composing of 16 ml cyclohexane and 4 ml methanol. And 10 mL BTP stock solution was quickly added into above-mixed solution to obtain the BTB/BTP stock solution. When 1 ml BTB/BTP stock solution was directly dropped onto the quartz substrate, and the organic core/mono-shell microwires were observed after the solvent completely evaporated. When 1 ml BTB/BTP stock solution was directly dropped onto the quartz substrate with 1 ml BTB stock solution, and the organic core/double-shell microwires were observed after the solvent completely evaporated. When 1 ml BTB/BTP stock solution was directly dropped onto the quartz substrate with 10 ml BTB stock solution, and 10 ml BTP stock solution were quickly added onto the quartz substrate at the same position. Finally, the organic core/double-shell microwires were observed after the solvent completely evaporated.

### Longitudinal epitaxial-growth mode for the organic segmented microwires

The sequential crystallisation process was utilised to fabricate the organic segmented microwires were obtained via a longitudinal epitaxial-growth process. In a typical experiment, 0.1 mmol BGP, *x* mmol TCNB, and *y* mmol TFP (*x* + *y* = 0.1) were first dissolved into 10 mL DCM at room temperature, forming the stock solution. Second, the 10 mL stock solution was added to the 20 mL mixed solution composing of 16 ml cyclohexane and 4 ml methanol. Then, 1 ml above-mixed solution was directly dropped onto the quartz substrate, and the organic segmented microwires were observed after the solvent completely evaporated.

## Supplementary information

Supplementary Information

## Data Availability

All data needed to evaluate the conclusions in the manuscript are presented herein and/or the Supplementary Materials. Additional data related to this study may be requested from the authors.
