# Supplementary material for: Organic superstructure microwires with hierarchical spatial organisation
Source: Nat Commun. 2021 Apr 15;12:2252. doi: 10.1038/s41467-021-22513-5 (PMC8050091; doi:10.1038/s41467-021-22513-5)
Supplement: Supplementary file 1 — Supplementary Information [file 41467_2021_22513_MOESM1_ESM.pdf]

Supplementary Information

Organic superstructure microwires with hierarchical spatial organisation

Ming-Peng Zhuo[1], Guang-Peng He[1], Xue-Dong Wang[1]*, Liang-Sheng Liao[1,2]*

[1]Institute of Functional Nano & Soft Materials (FUNSOM), Jiangsu Key Laboratory for Carbon-Based Functional Materials & Devices, Soochow University, 199 Ren'ai Road, Suzhou, Jiangsu 215123, P. R. China.

[2]Institute of Organic Optoelectronics, JITRI, Wujiang, Suzhou, Jiangsu 215215, P. R. China.

*E-mail: wangxuedong@suda.edu.cn (X.-D. Wang); lsliao@suda.edu.cn (L.-S. Liao)

Experimental details

1. Materials

Benzo[ghi]perylene (BGP, CAS: 191-24-2), 1,2,4,5-tetracyanobenzene (TCNB, CAS: 712-74-3), and tetrafluoroterephthalonitrile (TFP, CAS: 1835-49-0) were purchased from Sigma-Aldrich Co. The dichloromethane (DCM, analysis grade), methanol (analysis grade), *n*-hexane (analysis grade), cyclohexane (analysis grade) and ethanol (analysis grade) solvents were purchased from Sinopharm Group, China. In addition, all compounds and solvents were used without further treatment. The polytetrafluoroethylene filters (PTFE, Puradisc 25 TF, 0.1 μm) were bought from Whatman International Ltd.

2. Characterisations

The morphology and size of the organic micro/nanostructures were examined by emission scanning electron microscopy (FESEM, Carl Zeiss, Supra 55, Germany) with a 20 X-MaxN Energy Dispersive Spectrometer (EDS, Oxford Instruments, United Kingdom) dropping on an indium tin oxide (ITO) coated glass. TEM images were obtained by a transmission electron microscopy (TEM, FEI company, Tecnai G2 F20, United States). A drop of the solution was dropped on a carbon-coated copper grid, and evaporated. TEM measurement was performed at room temperature at an accelerating voltage of 100 kV. The X-ray diffraction (XRD) patterns were measured by a D/max 2400 X-ray diffractometer with Cu *K*α radiation (λ = 1.54050 Å) operated in the 2θ range from 5° to 30°, by using the samples on the quartz. Fluorescence images were recorded using a fluorescence optical microscope (Leica, DM4000M, Germany) with a spot-enhanced charge couple device (Diagnostic Instrument, Inc.). The excitation source is a mercury lamp equipped with a band-pass filter (330-380 nm for UV-light and 500~550 nm for green-light). The wavelengths of UV-light excitation and green-light excitation are the ordinary light. The samples were prepared by placing a drop of solution onto a cleaned quartz, and then evaporated at room temperature.

Micro-area photoluminescence (μ-PL) spectra were collected on a homemade optical microscopy. To measure the PL spectra of individual microplate, the micro/nanostructure was excited locally with a 375 nm laser focused down to the diffraction limit. The excitation laser was filtered with a 375 nm notch filter. The light was subsequently coupled to a grating spectrometer (Princeton Instrument, ARC-SP-2356) and recorded by a thermal-electrically cooled CCD (Princeton Instruments, PIX-256E). PL microscopy images were taken with an inverted microscope (Olympus, BX43).

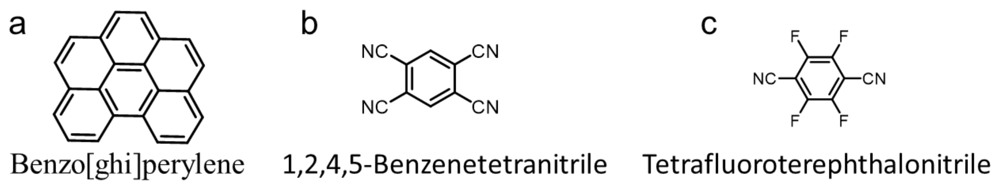

Supplementary Figure 1. The molecular structures of (a) benzo[ghi]perylene (BGP), (b) 1,2,4,5-Benzenetetranitrile (TCNB), and (c) tetrafluoroterephthalonitrile (TFP). The BTB cocrystal made up by BGP and TCNB. The BTB cocrystal made up by BGP and TFP.

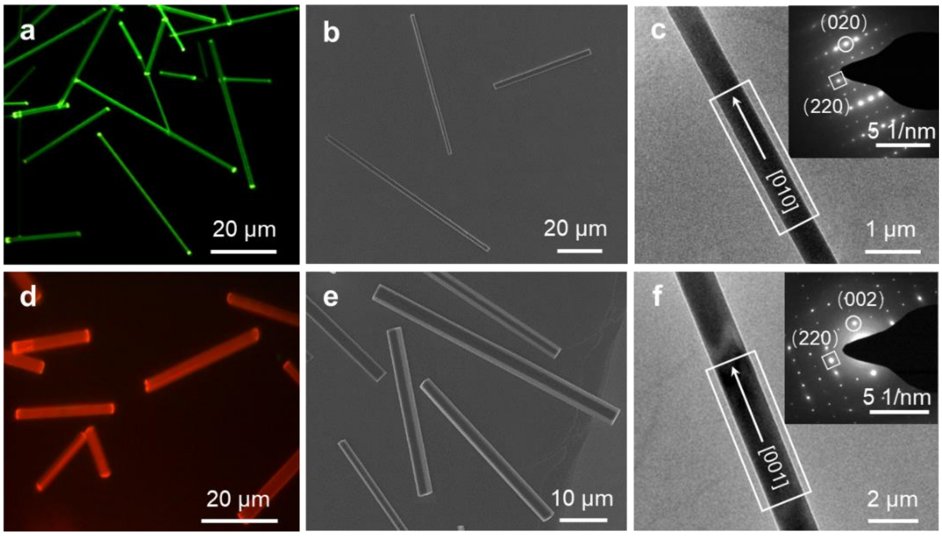

Supplementary Figure 2. Morphology of the organic microwires. The FM images of (a) BTP and (d) BTB microwires with the scale bars of 20 μm. The SEM images of (b) BTP and (e) BTB microwires. The scale bars are 20 and 10 μm, respectively. The TEM images of (c) BTP and (f) BTB microwires. The scale bars are 1 and 2 μm, respectively. Insets: the SAED patterns of these corresponding microcrystals.

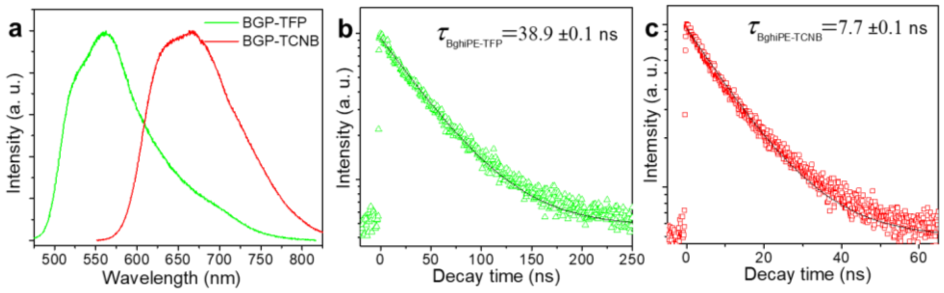

Supplementary Figure 3. Optical characterisations of the organic micro-/nano-crystals. (a) Spatially resolved PL spectra of BTP (green line) and BTB microwires (red line). PL decay curves of (b) BTP and (c) BTB microwires.

The modular structure of charge-transfer (CT) cocrystal demonstrates an outstanding advantage of facilely modifying and controlling over in both crystal-structure and optoelectronic features by tuning the CT interactions.[1, 2] The green-emissive BTP and the red-emissive BTB crystalline microwires with smooth surface were prepared by a solution-evaporation method,[3] as shown in Figures S1 and S2. The BTP microwires grow along the preferential [010] direction (Figure S1c), while BTB microwires grow along [001] direction (Figure S1f), which are coincident with their simulated growth morphology (Figure S3). Notably, the BTP and BTB cocrystals demonstrate similar structures and molecular parking mode (Figures S4 and S5), which prompted us to rationally design and fabricate the organic heterostructure microwires.

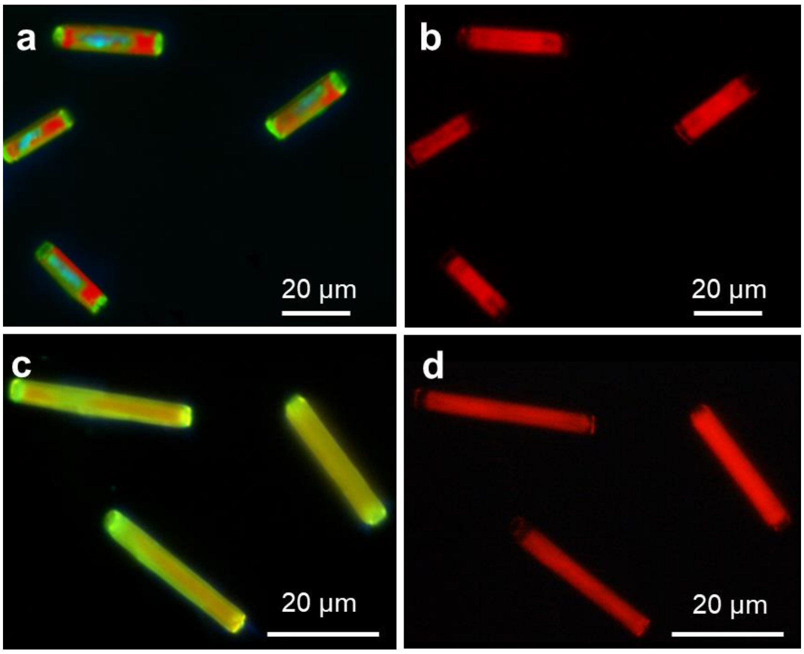

Supplementary Figure 4. Morphology of the organic BTB/BTP core/shell heterostructured microwires. (a-d) FM images of BTB/BTP core/shell microwires via a horizontal epitaxial-growth process. The organic core/shell microwires exited with (a, c) UV-light and (b, d) green-light. The scale bars are 20 μm.

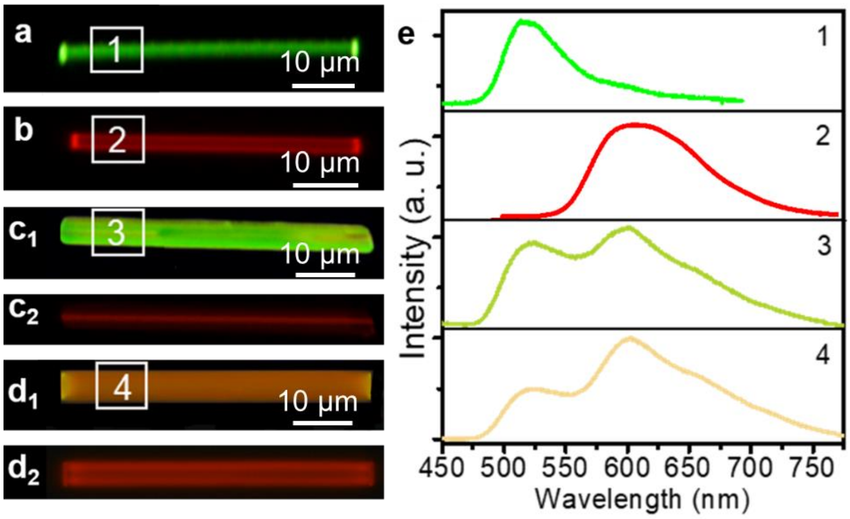

Supplementary Figure 5. Optical characterisations of the organic micro-/nano-crystals. FM images of the individual (a) BTP microwire, (b) BTB microwire, (c) BTB/BTP and (d) BTP/BTB core/shell heterostructured microwires with the scale bars of 10 μm. The microwire as shown in (a, b, c_1 and d_1) and (c_2 and d_2) were excited by UV-light and green-light. (e) Spatially resolved PL spectra collected from different locations marked in (a-d).

As shown in the Figures S4a and S4b, the FM images clearly suggests that the green-emissive BTP shell layer partially epitaxially grows on the surface of the BTB seed microwires, forming the defectiveness core/shell structure. The BTB/BTP core/shell microwires with a thick BTB shell layer demonstrated an intense yellow emission with excited by the UV-light as verified in the Figure S5c, which is attributed to a combination of the red-emission from BTB core microwire and green-emission form BTP shell layer. The BTB/BTP core/shell microwires only display red-emission from the BTB core excited by green-light (Figure S5d). The individual BTB/BTP core/shell microwires show an obviously recognisable microwires with intense red emission excited by green-light (Figure S5c_2), whose dimeter is smaller than that of the same microwires excited by the UV-light (Figure S5c_1). Combining the spatially resolved PL spectra of the BTB/BTP core/shell microwires (Figure S5e) including the green-emission (λ= 515 nm) from BTP cocrystal and red-emission (λ= 600 nm) from BTB cocrystal, it confirms the formation of BTB/BTP core/shell microwires via a horizontal epitaxial-growth in the multistep seeded growth method.[4, 5] Likewise, the

BTP/BTB core/shell microwires also present the yellow emission excited by the UV-light (Figures S4a and S5d$_1$) and the red-emission tube structure excited by the green-light (Figures S4a and S5d$_2$). Its spatially resolved PL spectra includes the green-emission (λ= 515 nm) and red-emission (λ= 600 nm), which indicates the formation core/shell microwires made up of the red-emission BTB shell layer and green-emission BTP core microwires.

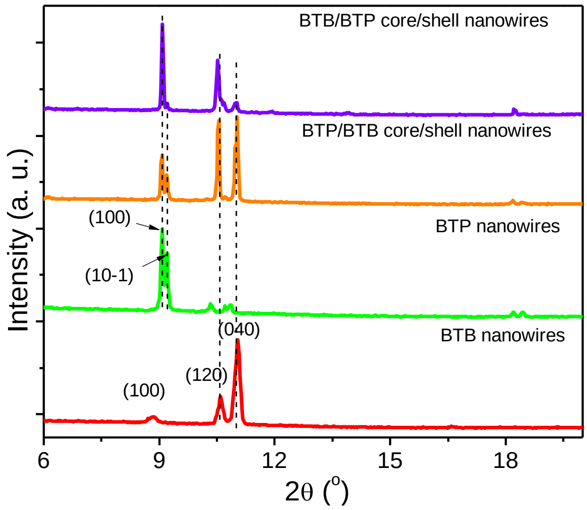

Supplementary Figure 6. Structure character of the organic core/shell microwires. XRD characterisations of BTP microwires (green line), BTB microwires (red line), BTP/BTB (orange line) and BTB/BTP (purple line) core/shell heterostructured microwires.

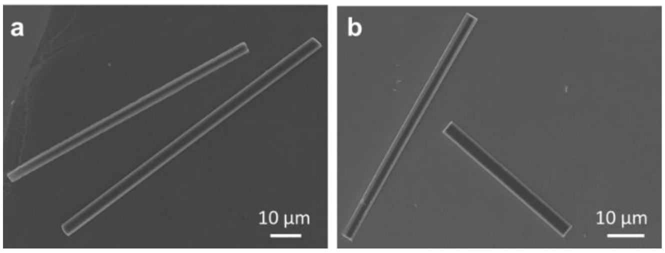

Supplementary Figure 7 Morphology of the organic core/shell microwires. SEM images of (a) BTP/BTB and (b) BTB/BTP core/shell microwires. The scale bars are 10 µm.

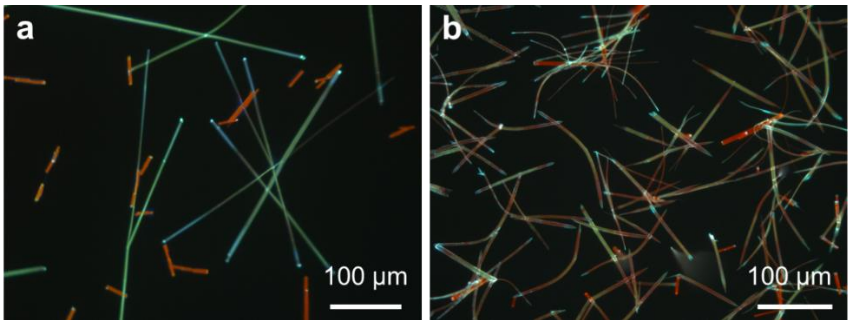

Supplementary Figure 8. The influence of solvent/temperature on the growth of core-shell. FM images of organic microwires prepared by (a) adding the stock solution composing BTP and BTB at 25℃ to the pure cyclohexane and (b) adding the stock solution at 40℃ to the mixed solution composing of cyclohexane and methanol (the volume ratio: $V_{cyclohexane}$:$V_{methanol}$ = 4:1). The scale bars are 100 μm.

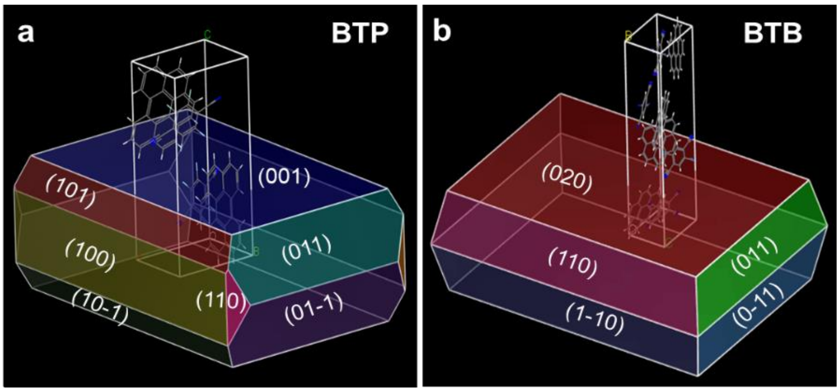

Supplementary Figure 9. Predicted growth morphology of (a) BTP and (b) BTB cocrystals.

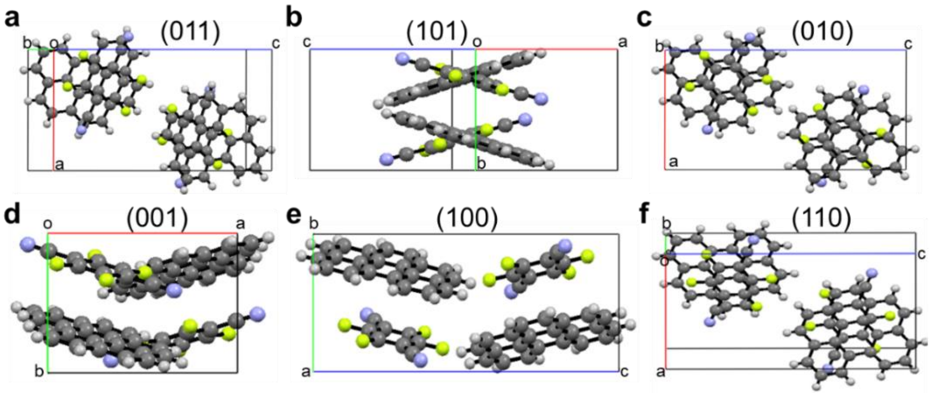

Supplementary Figure 10. Unit cell structure of BTP crystals. Molecular packing arrangement in (a) (011), (b) (101), (c) (010), (d) (001), (e) (100) and (f) (110) planes.

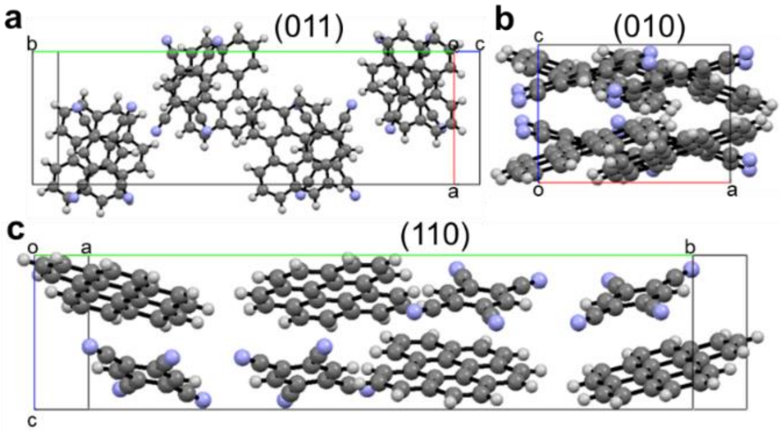

Supplementary Figure 11. Unit cell structure of BTB crystals. Molecular packing arrangement of (a) (011), (b) (010) and (c) (110) planes.

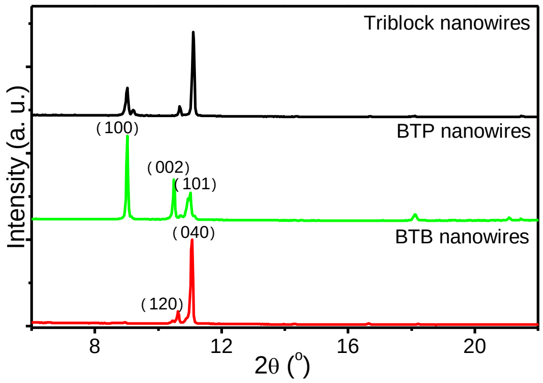

Supplementary Figure 12. Structural characterisation of the organic triblock microwires. XRD characterisations of BTP microwires (green line), BTB microwires (red line), and triblock microwires (black line).

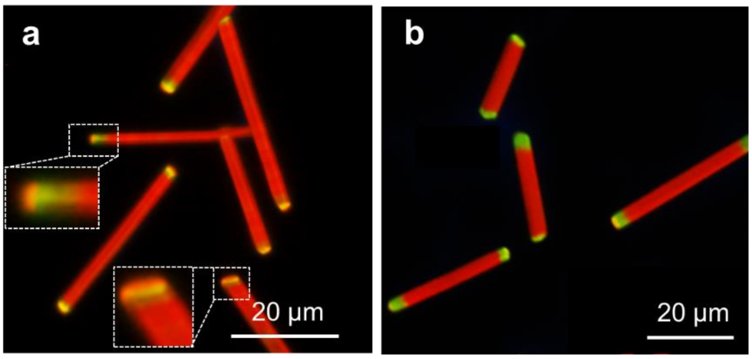

Supplementary Figure 13. Morphology of the organic triple-block microwires. FM images of organic triblock microwires with η_{TFP} of (a) 12% and (b) 17% with scale bars of 20 μm.

The green-emissive block of organic triblock microwires obtained at η_{TFP} of 15% (Figure 4f) is longer than that obtained at η_{TFP} of 17% (Figure S13a), is shorter than that obtained at η_{TFP} of 12% (Figure S13b). It indicates that the η_{TFP} can be used to rationally turn the length ratio of the functional block in the organic triblock microwires. Remarkably, it was found that there is a very short red block appear on the tips of the organic triple-block microwires obtained at η_{TFP} of 12%, as verified by the marked tips in the Figure S13.

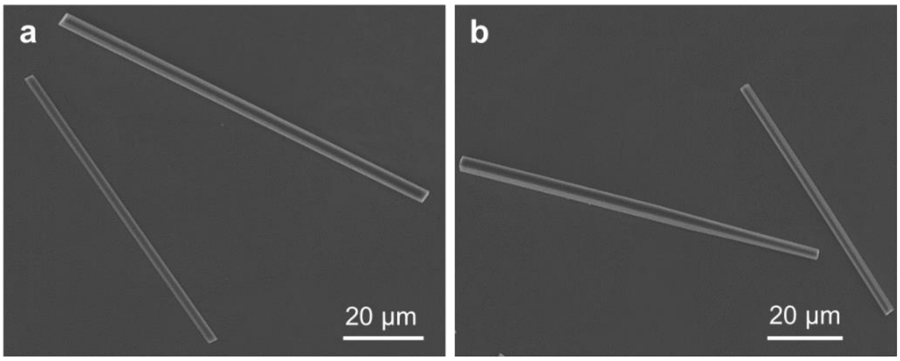

Supplementary Figure 14. Morphology of organic segmented microwires. SEM images of (a) Triple-block and (b) Quintuple-block microwires. Scale bars are 20 μm.

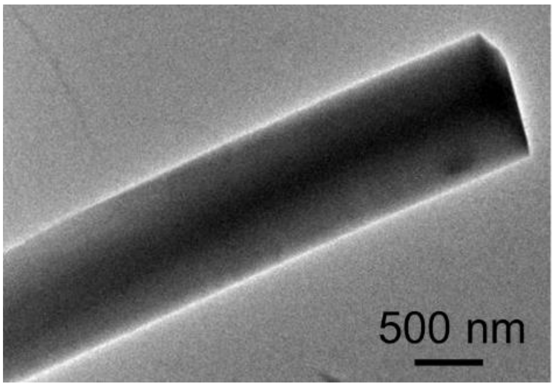

Supplementary Figure 15. Morphology of organic segmented microwires. A high-resolution TEM image of an individual organic triple-block microwire with a scale bar of 500 nm.

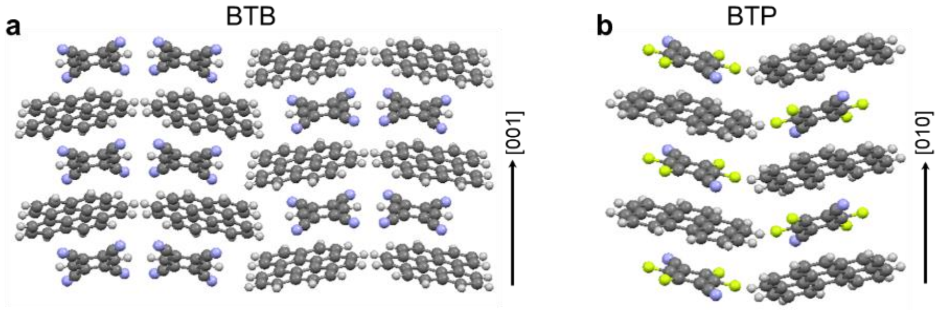

Supplementary Figure 16. Molecular packing arrangement of (a) the *ab* plane in BTB cocrystal along *c*-axis and (b) the *ac* plane in BTP cocrystal along the *b*-axis.

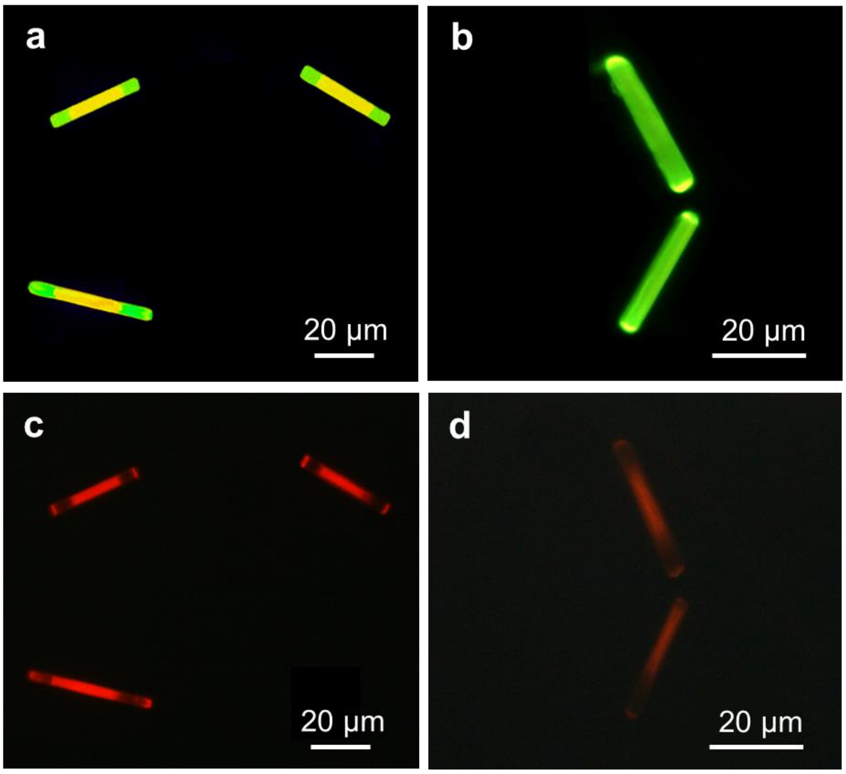

Supplementary Figure 17. Morphology of the organic superstructure microwires. FM images of segmented-core/shell type-I organic superstructure microwires with low concentration of BTP precursor excited by (b) UV-light and (d) green-light. FM images organic superstructure microwires with high concentration of BTP precursor by (a) UV-light and (c) green-light. Scale bars are 20 μm.

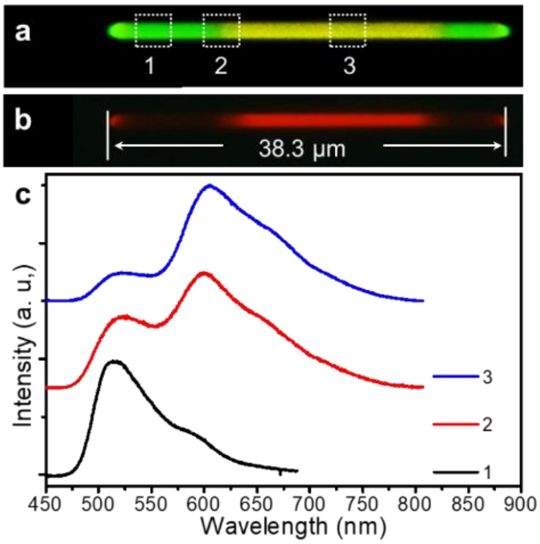

Supplementary Figure 18. Optical characterisations of the organic superstructure microwires. FM images of the segmented-core/shell type-I organic superstructure microwires excited by (a) UV-light and (b) green-light. (c) Spatially resolved PL spectra collected from different locations marked in (a).

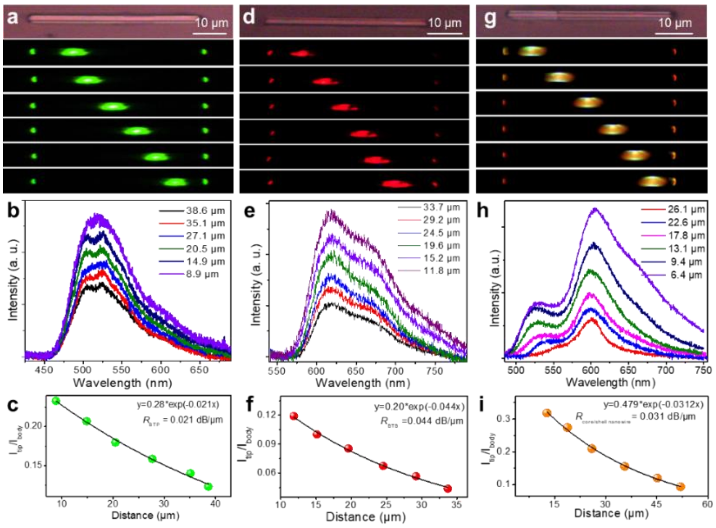

Supplementary Figure 19. Optical waveguide properties of the organic microwires. (a) FM images obtained from an individual BTP microwire by exciting with a laser beam (λ = 375 nm) at different positions with a scale bar of 10 μm. (b) Corresponding spatially resolved PL spectra in (a) with different separation distances *d*. (c) Ratios of intensity I_{tip}/I_{body} against distance *d*. Curves were fitted by an exponential decay function $I_{tip}/I_{body} = A \exp(-Rd)$.[6] (d-i) Optical waveguide characterisation of (d-f) BTB microwires, and (g-i) BTB/BTP core/shell microwires.

Figures S18a, S18d and S18g display the microarea FM images obtained from a BTP microwire (length; 50.1 μm), BTP microwire (length; 46.3 μm) and BTB/BTP core/shell microwire (length; 58.6 μm), respectively, by accurately shifting the excitation laser spots (diameter ~2 μm, λ=375 nm) along the length of one dimensional structure. Figure S18b clarifies the spatially resolved PL spectra detected from the tip of an individual BTP microwire based on the various photon propagation distance *d*. It revealed an obvious decrease in PL intensity, which is positive with the photon propagation distance *d*. The PL intensities at the excited site along the body of the prepared 1D microwire (I_{body}) and at the emitting tip (I_{tip}) were recorded, and the ratio I_{tip}/I_{body} shows a single-exponential decay against the propagation distance *d* (Figure S18c), which indicates an active nature of the optical waveguide.[3, 7] The optical-loss coefficient (*R*) was calculated by single-exponential fitting I_{tip}/I_{body} = A exp(−*Rd*), where *d* is photon propagation distance from the excited site to the emitting tip.[6] Accordingly, the optical-loss coefficient of BTP (R_{BTP}) and BTB (R_{BTB}) microwire is 0.021 and 0.044 dB μm^{-1} at 515 and 600 nm, respectively, which are comparable with that of these previously reported inorganic/organic optical waveguides.[8-10] High crystallinity and smooth surface play crucial roles in the low optical loss in BTP and BTB organic microwires. They can effectively reduce optical losses caused by scattering. Notably, the obvious emission shift is absent when increasing the propagation distance in BTB/BTP core/shell organic microwires as shown in Figures S18g and S18h. The corresponding optical-loss coefficient is 0.031 dB μm^{-1} (Figure S18i), which is due to photon confinement effect and the efficient energy transfer process in core/shell structure.[4]

Supplementary Table 1. Intermolecular interaction and lattice energy intensity. The DFT calculations for CT interaction: $E_{\mathrm{BTB}}^{\mathrm{interaction}}$ = -3.14 kcal mol^{-1} and $E_{\mathrm{BTP}}^{\mathrm{interaction}}$ = -2.84 kcal mol^{-1}; lattice energy intensity: $E_{\mathrm{BTB}}^{\mathrm{lattice}}$ = -66.57 kcal mol^{-1} and $E_{\mathrm{BTP}}^{\mathrm{lattice}}$ = -60.15 kcal mol^{-1}.

Name	Molecular structure	Lattice energy (kcal mol^{-1})	CT interaction (kcal mol^{-1})
BTP	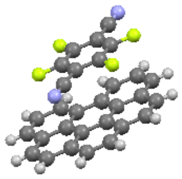	-60.15	-66.57
BTB	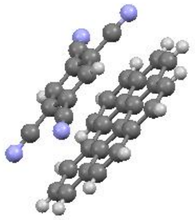	-2.84	-3.14

The interaction energy between organic molecules were performed by using Gaussian 09 software package[11] with 6-31+G(d,p) basis set and B3lyp level of theory[12] after structure optimisation in the vacuum. It was conducted according to the following equation considering the Basis Set Superposition Error (BSSE) correction: $E_{\mathrm{int}} = E_{\mathrm{tot}} - (E_1 + E_2) + E_{\mathrm{bsse}}$, where E_{int} is the interaction energy between the two molecules, E_1 and E_2 is the energy of single molecules and E_{bsse} are the energies of BEES correction.

Density-functional theory (DFT) computations were performed using the Dmol^3 software package based on the linear combination of atomic orbitals (LCAO) method. Electron-ion interactions were described using the all electron (AE) method. A double numerical polarised (DNP) basis set was employed to expand the wave functions with an orbital cutoff of 4.0 Å. For the electron-electron exchange and correlation interactions, the functional parametrised by Perdew-Burke-Ernzerhof (PBE), a form of the general gradient approximation (GGA), was used throughout. The vander Waals interaction was described using the DFT-D2 method that proposed by Grimme. During the geometry optimisations, atomic positions and the lattice parameters were allowed to relax. In this work, the Brillouin-zone integrations were conducted using Monkhorst-

Pack (MP) grids of special points. A *k*-point sets with a separation of 0.06 Å^{-1} was used for all the model cells. The convergence criterion for the electronic self-consistent field (SCF) loop was set to 10^{-6}. The atomic structures were optimised until the residual forces were below 0.002 Ha Å^{-1}.

Supplementary Table 2. Attachment energies ($E^{attch}_{\{hkl\}s}$) and surface free energies ($E^{surf}_{\{hkl\}s}$) of various crystal facets calculated by using the material studio package.

{hkl}	*d* (Å)	Surface area (%)	BTB	
			$E^{attch}_{\{hkl\}s}$ (kcal/mol)	$E^{surf}_{\{hkl\}s}$ (kcal/mol)
{020}s	15.74	53.7	-34.29	8.57
{110}s	9.22	27.3	-79.82	40.02
{011}s	6.96	9.5	-111.71	56.41
{hkl}	*d* (Å)	Surface area (%)	BTP	
			$E^{attch}_{\{hkl\}s}$ (kcal/mol)	$E^{surf}_{\{hkl\}s}$ (kcal/mol)
{001}s	16.45	49.4	-17.68	8.84
{100}s	9.15	15.9	-36.20	18.16
{110}s	5.57	0.6	-66.42	34.85
{011}s	6.46	9.4	-55.79	28.30
{101}s	8.00	8.2	-36.80	17.47
{10-1}s	8.00	6.5	-35.89	18.47
{020}s	3.51	3.0	-74.25	25.21

Supplementary References

1. Zhang, J.; Xu, W.; Sheng, P.; Zhao, G. & Zhu D. Organic Donor-Acceptor Complexes as Novel Organic Semiconductors. *Acc Chem. Res*. **50**, 1654-1662 (2017).
2. Huang, Y.; Wang, Z.; Chen, Z. & Zhang Q. Towards Emerging Properties and Applications of Organic Cocrystals Beyond Electrical Conductivities and Field-Effect Transistors (FETs). *Angew. Chem. Int. Ed*. **58**, 2-18 (2019).
3. Zhuo, M. P; Tao, Y. C.; Wang, X. D.; Wu, Y.; Chen, S.; Liao, L. S.; *et al.* 2D

Organic Photonics: An Asymmetric Optical Waveguide in Self-Assembled Halogen-Bonded Cocrystals. *Angew. Chem. Int. Ed.* **130**, 11470-11474 (2018).

4. Zhuo, M. P.; Fei, X. Y.; Tao, Y. C.; Fan, J.; Wang, X. D.; Xie, W. F. *et al.* In Situ Construction of One-Dimensional Component-Interchange Organic Core/Shell Microrods for Multicolor Continuous-Variable Optical Waveguide. *ACS Appl. Mater. Interfaces* **11**, 5298-5305 (2019).
5. Zhuo, M. P.; Wu, J. J.; Wang, X. D.; Tao, Y. C.; Yuan, Y.; Liao, L. S. Hierarchical self-assembly of organic heterostructure microwires. *Nat. Commun.* **10**, 3839 (2019).
6. Yao, W.; Yan, Y.; Xue, L.; Zhang, C.; Li, G.; Zheng, Q. *et al.* Controlling the Structures and Photonic Properties of Organic Nanomaterials by Molecular Design. *Angew. Chem. Int. Ed.* **52**, 8713-8717 (2013).
7. Wang, X.; Zhou, Y.; Lei, T.; Hu, N.; Chen, E. Q.; Pei J. Structural-Property Relationship in Pyrazino[2,3-g]quinoxaline Derivatives: Morphology, Photophysical, and Waveguide Properties. *Chem. Mater.*, **22**, 3735-3745 (2010).
8. Qu, G.; Hu, Z.; Wang, Y.; Yang, Q.; Tong, L. Synthesis of Optical-Quality Single-Crystal β-BaB_2O_4 Microwires and Microwires. *Adv. Funct. Mater*. **23**, 1232-1237 (2012).
9. Yan, B.; Liao, L.; You, Y.; Xu, X.; Zheng, Z.; Shen, Z. *et al.* Single-Crystalline V_2O_5 Ultralong Nanoribbon Waveguides. *Adv. Mater.* **21**, 2436-2440 (2009).
10. Zhang, Q.; Shang, Q.; Shi, J.; Chen, J.; Wang, R.; Mi, Y. *et al.* Wavelength Tunable Plasmonic Lasers Based on Intrinsic Self-Absorption of Gain Material. *ACS Photonics*, **4**, 2789-2796 (2017).
11. Frisch, M. J.; Trucks, G. W.; Schlegel, H. B.; Scuseria, G. E.; Robb, M. A.; Cheeseman, J. R.; Scalmani, G.; Barone, V.; Mennucci, B.; Petersson, G. A.; Nakatsuji, H.; Caricato, M.; Li, X.; Hratchian, H. P. Izmaylov, ; A. F.; Bloino, J.; Zheng, G.; Sonnenberg, J. L.; Hada, M.; Ehara, M.; Toyota, K.; Fukuda, R.; Hasegawa, J.; Ishida, M.; Nakajima, T.; Honda, Y.; Kitao, O.; Nakai, H.; Vreven, T.; Montgomery, J. A.; Jr., J. E. P.; Ogliaro, F.; Bearpark, M.; Heyd, J. J.; Brothers, E.; Kudin, K. N.; Staroverov, V. N.; Kobayashi, R.; Normand, J.;

Raghavachari, K.; Rendell, A.; Burant, J. C.; Iyengar, S. S.; Tomasi, J.; Cossi, M.; Rega, N.; Millam, J. M.; Klene, M.; Knox, J. E.; Cross, J. B.; Bakken, V.; Adamo, C.; Jaramillo, J.; Gomperts, R.;; Stratmann, R. E. Yazyev, O.; Austin, A. J.; Cammi, R.; Pomelli, C.; Ochterski, J. W.; Martin, R. L.; Morokuma, K.; Zakrzewski, V. G.; Voth, G. A.; Salvador, P.; Dannenberg, J. J.; Dapprich, S.; Daniels, A. D.; Farkas, Ö.; Foresman, J. B.; Ortiz, J. V.; Cioslowski J. and Fox, D. J. Gaussian 09 A02.

12. Hehre, W. J.; Ditchfield, R.; Pople, J. A., Self-Consistent Molecular Orbital Methods. XII. Further Extensions of Gaussian-Type Basis Sets for Use in Molecular Orbital Studies of Organic Molecules. *J Chem Phys* **56**, 2257-2261 (1972).
